# Maternal Th17/Treg Cytokines and Small Extracellular Vesicles in Plasma as Potential Biomarkers for Preeclampsia

**DOI:** 10.7150/ijms.71047

**Published:** 2022-09-25

**Authors:** Han-Yu Chen, Xin-Yu Wang, Ke-Ming Lu, Chen-Hsiang Yu, Mei-Tsz Su, Lin Kang, Keng-Fu Hsu, Po-Fan Chen, Sheng-Hsiang Lin

**Affiliations:** 1 Institute of Clinical Medicine, College of Medicine, National Cheng Kung University, Tainan, Taiwan.; 2 Department of Laboratory Medicine, Kaohsiung Chang Gung Memorial Hospital and Chang Gung University College of Medicine, Kaohsiung, Taiwan.; 3 Department of Obstetrics and Gynecology, National Cheng Kung University Hospital, College of Medicine, National Cheng Kung University, Tainan, Taiwan.; 4 Department of Public Health, College of Medicine, National Cheng Kung University, Tainan, Taiwan.; 5 Biostatistics Consulting Center, National Cheng Kung University Hospital, College of Medicine, National Cheng Kung University, Tainan, Taiwan.

**Keywords:** preeclampsia, Th17, Treg, cytokine, small extracellular vesicles, predictive biomarker, discriminant analysis, machine learning, support vector machine

## Abstract

Preeclampsia is one of the most serious pregnancy complications. It may be caused by immunological changes in the early placental microenvironment. The contents of small EVs may serve as biomarkers of pregnancy complications. Evidence suggests that the balance between T helper 17 (Th17) and regulatory T (Treg) cells are critical for preventing preeclampsia. The study recruited 39 pregnant women with preeclampsia and 127 healthy pregnant women. We assessed the levels of both Th17 and Treg cytokines (IL-10, IL-17, IL-21, IL-22, and TGF-β) in their plasma and small EVs. We found significant differences in the levels of all cytokines in the plasma between the two groups during the second trimester. We also observed significant differences between the two groups in the levels of EV-encapsulated cytokines IL-21, IL-22, and TGF-β, as well as in total small EVs, during the second trimester. The ROC analysis showed that the classification efficiency (AUC) of TGF-β in small EVs was 0.81. TGF-β had the best discriminant ability of all the single EV biomarkers tested, the cross-validation of the accuracy was 0.89. Th17 and Treg cytokines in plasma and small EVs may contribute to maternal immune activation and clarify the potential mechanisms of small EVs and cytokines in preeclampsia.

## Introduction

Preeclampsia affects 2-8% of pregnancies and is characterized by new-onset hypertension combined with proteinuria after 20 weeks of gestation 1. It is a multisystemic disorder that targets several organs and results in severe complications, causing maternal and neonatal morbidity and mortality 2, 3. Various reports indicate that placental dysfunction participates in the induction of preeclampsia 4, 5. One pathway involves abnormal placentation, resulting in placental ischemia and inflammation, along with the release of pro-inflammatory cytokines into the maternal circulation. These factors may lead to maternal endothelial dysfunction in early gestation 6.

Small extracellular vesicles (EVs) are a group of heterogeneous cell-derived membrane vesicles, including exosomes, microvesicles, and apoptotic bodies 7. Some evidence suggests that organs or cells secrete small extracellular vesicles (EVs) for cell communication and immune adaptation during pregnancy 8. Small EVs (diameter of 50-150 nm) harbor the proteins, RNA, and DNA of the origin cell, and are detected in various bodily fluids, including blood 9. They may contribute to the systemic inflammatory response observed in pregnant women with preeclampsia 10. Inflammatory cytokines are generally thought to exert biological influence as soluble molecules that are encapsulated within small EVs 11.

T helper 17 (Th17) and regulatory T (Treg) cells are part of the complex immune machinery 12. Th17 cells are key effector T cells in the induction of inflammatory responses, since they produce pro-inflammatory cytokines such as IL‐17, IL-21, and IL-22 13. On the other hand, Treg cells produce transforming growth factor beta (TGF-β) and IL-10, and Treg cytokines play central roles in immunological regulation and tolerance 14. The mechanism underlying the role of an imbalance between Th17 and Treg cells in preeclampsia remains unclear, although significant relationships between peripheral blood immune effectors and the development of preeclampsia were observed.

The elevated levels of pro-inflammatory cytokines in the maternal circulation system may play a central role in excessive inflammatory response and endothelial dysfunction. It is thought that small EVs may support the exaggerated inflammatory state observed in preeclampsia. Recent studies have demonstrated a shift in the balance of Th17 and Treg cytokines in patients with preeclampsia. We hypothesized that the imbalance in Th17, Treg cytokines and maternal small EVs in the plasma, are involved in immunoregulation and may be potential biomarkers for preeclampsia. Our specific aims were: (1) To investigate the association of plasma Th17 and Treg cytokines in preeclampsia patients and healthy controls; (2) To evaluate the association of total small EVs, placenta-derived small EVs, and EV-encapsulated cytokines in preeclampsia patients and healthy controls; and (3) To assess whether these plasma and EV-encapsulated cytokines could be used as biomarkers for preeclampsia.

## Materials and methods

### Patients

This study was designed as a prospective cohort study, including pregnant women in their early pregnancy. Then tracked to production and investigated disease (i.e., preeclampsia) happen or not. Pregnant women were recruited at National Cheng Kung University hospital (NCKUH) from October 2015 to February 2019. We then collected the informed consent, questionnaires and their residual blood samples. The definition of pregnancy trimesters in this study: the first trimester was 0-13 weeks; the second trimester was 14-27 weeks; the third trimester was 28-42 weeks to delivery. Participants' samples in first trimester were collected between 12-13weeks, participants' samples in second trimester were collected between 14-27 weeks and participants' samples in third trimester were collected between 28-39 weeks. The cases were included if they met the criteria of American College of Obstetricians and Gynecologists' clinical guidelines for preeclampsia (new-onset hypertension (blood pressure ≥ 140/90 mmHg on two occasions separated by at least 4 h) and proteinuria (> 300 mg/24 h or protein/creatinine ratio>0.30) after 20 weeks of gestation). We got diagnosis data from medical records. Controls were healthy pregnant individuals without other major obstetric complications. Those pregnancies with preeclampsia onset before collecting blood specimen in second and third trimester and the women with other major obstetric complications were excluded. Finally, we totally included 39 pregnant women with preeclampsia and 127 healthy pregnant women as controls. The study was approved by the Institutional Review Board at NCKUH (IRB numbers: B-ER-103-418 and B-BR-106-003-T).

### Measurement the level of cytokines in plasma and small EVs

Total small EVs were isolated from the plasma using an qEV size exclusion columns (35nm series, Izon Science). Purification of small EVs using qEV size exclusion columns was performed according to instructions provided by the manufacturer. Plasma samples (150 μL) were first overlaid on qEV size exclusion columns followed by elution with PBS, finally 200 μL fractions were collected. Small EV was analysed by the tunable resistive pulse sensing (TRPS) using NP200 Nanopore (qNano, Izon Science Ltd) to get the concentration and size distribution of particles. Carboxylated polystyrene beads (200nm) were used to calibrate the concentration and size. Transmission electron microscopy (TEM) was also used to visualize the morphology of small EV. We added 50-100 μl small EVs for ELISA experiments (CD63 for 100 μl small EVs; PLAP for 50 μl small EVs) according to the instructions of the protocol. The concentrations of total and placental small EVs were quantified using CD63 (MyBio-source, USA) and placental alkaline phosphatase (PLAP) (ELISA Kit for Alkaline Phosphatase, Placental, MyBio-source, USA) enzyme-linked immunosorbent assay (ELISA) kits. CD63 is a common small EVs surface marker and PLAP is a syncytiotrophoblast-specific marker, so small EVs derived from the placenta are positive for PLAP.

Plasma samples were prepared by brief centrifugation of whole blood samples and stored at -80 °C until analysis. Small EVs samples were prepared by adding 5ml radioimmunoprecipitation assay (RIPA) buffer (Thermo Fisher Scientific, USA) with 50ul protease inhibitor (Thermo Fisher Scientific, USA) directly to small EVs suspended in PBS to lyse the small EVs membrane and solubilize the proteins. We added 100 μl small EVs for ELISA experiments (IL-17, IL-21, IL-22, IL-10, and TGF-β for 100 μl small EVs) according to the instructions of the protocol. We determined the plasma and levels of EV-encapsulated Th17 (IL-17 (Human IL-17A ELISA MAX Deluxe, BioLegend, USA), IL-21 (IL-21 ELISA Ready-Set-Go Sets, Thermo Fisher Scientific, USA), and IL-22 (IL-22 ELISA Ready-Set-Go Sets, Thermo Fisher Scientific, USA)) and Treg (IL-10 (IL-10 ELISA Ready-Set-Go Sets, Thermo Fisher Scientific, USA) and TGF-β (Human/Mouse TGF beta ELISA Ready-Set-Go Sets, Thermo Fisher Scientific, USA)) cytokines using the ELISA kits according to the instructions provided by the manufacturer. The plasma and small EVs samples from both groups were assayed using the same ELISA plate to minimize experimental variation.

### Statistical analysis

All analyses were performed using SAS version 9.4 (SAS Institute, Cary, NC, USA) and R version 3.6.1 (The R Foundation for Statistical Computing, Vienna, Austria; https://www.r-project.org/). We compared maternal demographic characteristics and immunological and EV biomarkers between the two groups. For categorical variables, Pearson's chi-squared test or Fisher's exact test was used to determine significant differences. For continuous variables, Student's t-test or the Wilcoxon rank-sum test was used to determine the significance of differences between the two groups. The values for each biomarker were divided into quartiles and included in the logistic regression model as continuous variables. A P-value of < 0.05 was considered statistically significant.

Receiver operating characteristic (ROC) curves were constructed using logistic regressions to produce plots of the percentage of true positives (sensitivity) and false positives (1 - specificity) for the multivariate immunological levels in preeclampsia patients and healthy controls. The area under the ROC curve (AUC) was calculated as the predictive value of using plasma and EV biomarker levels to differentiate between preeclampsia patients and healthy controls. In addition, a type of machine learning algorithm, support vector machine (SVM), was used to assess classification efficiency and establish the prediction models for disease status based on multiple cytokines 15. An SVM constructs a hyperplane or set of hyperplanes in a high- or infinite-dimensional space, which can be used for classification. Thus, a good separation is achieved by the hyperplane that has the largest distance from the nearest training datapoint in the functional margin. The SVM models were built and run with leave-one-out cross-validation to prevent overfitting of the predictive signature. In this study, the radial kernel was used to estimate the optimal model parameters 16. Accuracy, sensitivity, and specificity were evaluated using the classifier.

## Results

### Demographic and clinical characteristics of the study participants

There were significant differences in body mass index (BMI), blood pressure, and parity between the preeclampsia patients and healthy controls: the preeclampsia patients had a higher mean BMI, they had a higher incidence of chronic hypertension, and they tended to have earlier deliveries (Table 1). Indeed, their incidence of preterm delivery was significantly higher. Their infants also had a significantly lower birth weight and height.

### Plasma cytokine levels in preeclampsia patients and healthy controls

The plasma levels of all five cytokines were significantly higher in the preeclampsia patients during the second trimester, and also of all except TGF-β during the third trimester (Table S1). Dot plot for the sample data after ELISA analysis of Th17 and Treg cytokines between PE patients and healthy controls were showed in Figure S1 and S2. Although there was no significant difference in the pro- to anti-inflammatory plasma cytokine ratios between the study groups (Table S2), the values of the results nevertheless indicated higher pro-inflammatory to anti-inflammatory cytokine ratios in the preeclampsia patients (Figure S5).

### EV biomarker levels in preeclampsia patients and healthy controls

**Figure 1:**
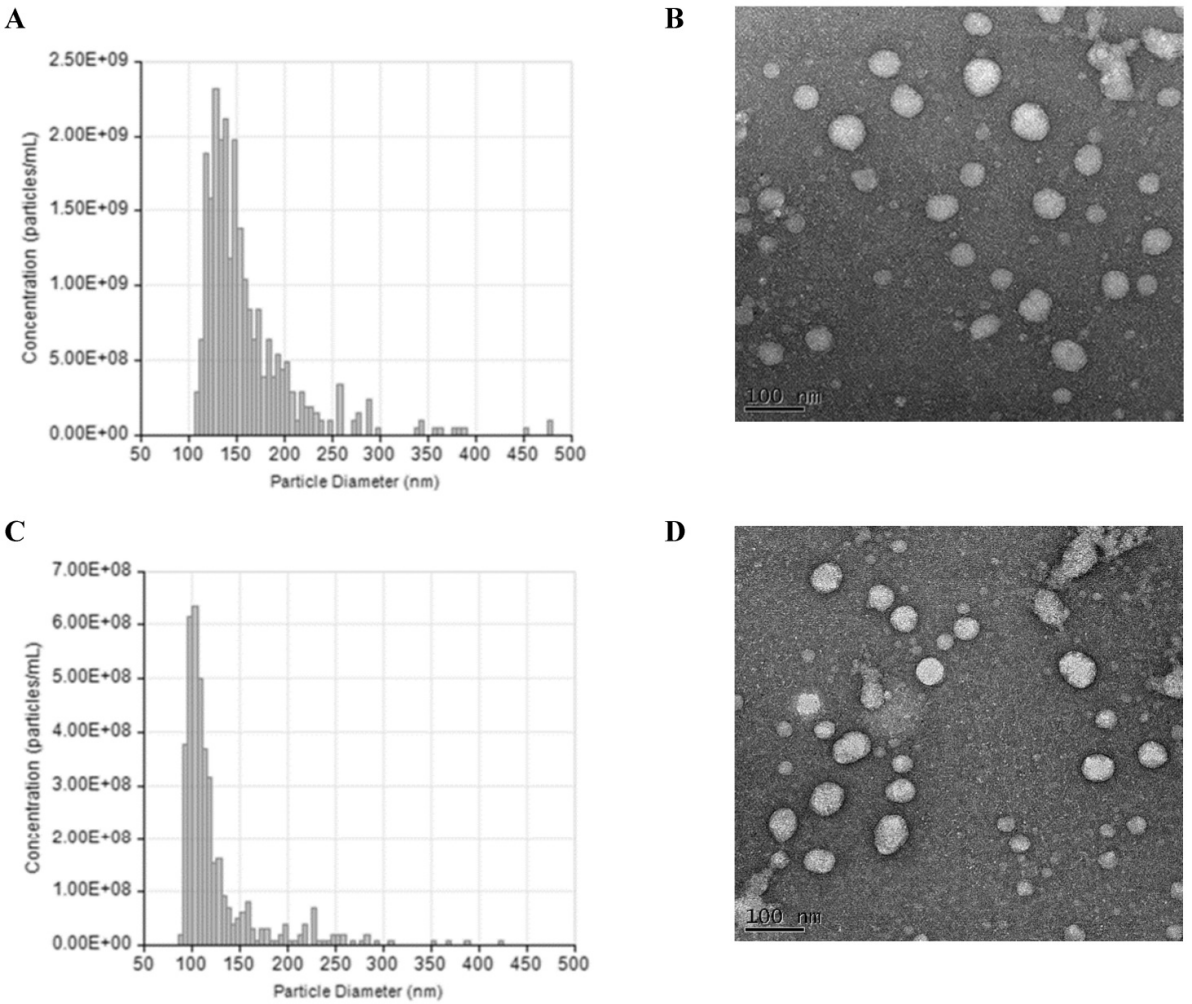
** Characterization of small extracellular vesicles (EVs). (A)** The result of tunable resistive pulse sensing (TRPS) analysis show the size and concentration distribution of particles consistent with the size range of small EVs in preeclampsia (PE). **(B)** Imaging of small extracellular vesicles (EVs) by Transmission Electron Microscopy in preeclampsia (PE). **(C)** The result of tunable resistive pulse sensing (TRPS) analysis show the size and concentration distribution of particles consistent with the size range of small EVs in healthy controls (HC). **(D)** Imaging of small extracellular vesicles (EVs) by Transmission Electron Microscopy in healthy controls (HC).

The NTA and TEM data of small EVs in both groups were showed in Figure 1. According to the analysis, the average concentration of EVs was about 6.8e+08 particles/ml. During the second trimester, the total small EVs concentration and levels of three EV-encapsulated cytokines (IL-21, IL-22, and TGF-β) were significantly higher in the preeclampsia patients than in the healthy controls (Table 2). During the third trimester, the concentration of placental small EVs and levels of two EV-encapsulated cytokines (IL21 and IL-22) were significantly higher in the preeclampsia patients. Dot plot for the total and placental-derived small EVs concentration and exosomal levels of Th17 and Treg cytokines between PE patients and healthy controls were showed in Figure S3 and S4. There were also significant differences in the IL-17/TGF-β, IL-21/IL-10, IL-21/TGF-β, and IL-22/TGF-β ratios between the two groups (Table S3).

### Efficiency of plasma and EV biomarkers for classifying preeclampsia patients

During the second trimester, plasma IL-22 levels had the highest AUC, which was 0.67, of all the candidate plasma biomarkers when considered individually (Table S4). When they were combined, the combination of IL-21 and IL-22 performed even better (AUC = 0.71), and similarly to the combination of all three of the plasma Th17 cytokines (IL-17, IL-21 and IL22) whose AUC was 0.72.

The total small EVs concentration (AUC = 0.77) and placental small EVs concentration (AUC = 0.71) during the second trimester demonstrated a good classification efficiency (Table 3). Two EV-encapsulated cytokines also performed well during the second trimester (IL-21: AUC = 0.76; TGF-β: AUC = 0.81). The combination of total small EVs concentration and EV-encapsulated TGF-β levels (AUC = 0.89), as did the placental small EVs concentration combined with EV-encapsulated TGF-β (AUC = 0.88) and the combination of IL-17, IL-21, and TGF-β (AUC = 0.88).

### Development and cross-validation of the cytokine signature for discriminating between healthy controls and preeclampsia patients

Using the SVM, we evaluated the performance of a single plasma cytokine as a predictor, and found, after cross-validation, that IL-22 (cross-validation accuracy = 75%) and TGF-β (cross-validation accuracy = 77%) displayed relatively high accuracy in discriminating between healthy controls and the patients with preeclampsia (Table S4). However, the ability of the plasma cytokine ratios to identify individuals with preeclampsia was not strong (Table S5).

In terms of the EV biomarkers, total small EVs concentration (cross-validation accuracy = 71%) and placental small EVs concentration (cross-validation accuracy = 74%) both exhibited strong classification ability during the second trimester (Table 3). The same was true for EV-encapsulated IL-22 (cross-validation accuracy = 74%) and TGF-β (cross-validation accuracy = 89%). The results were even stronger for the combination of placental small EVs and EV-encapsulated TGF-β (cross-validation accuracy = 90%). The classification model also performed well using the combination of EV-encapsulated IL-21 and TGF-β (cross-validation accuracy = 86%) or of IL-17, IL-21, and TGF-β (cross-validation accuracy = 85%). We also conducted an SVM analysis of the EV-encapsulated cytokine ratios, and found that the classification ability of the combinations EV-encapsulated IL-17/TGF-β, IL-21/TGF-β, and IL-22/TGF-β was good (Table S6). Lower ratios of EV-encapsulated IL-17/TGF-β and IL-22/TGF-β would thus be associated with an increasing risk of preeclampsia.

We also used the second trimester and third trimester data of the pregnant women and merged a small part of the recorded data from the first trimester to create generalized additive mixed models (GAMMs). GAMMs as an extension of generalized linear mixed models makes it possible to account for temporal autocorrelational structure in experimental data and assessed the pattern of TGF-β level, which has the highest accuracy, in EV markers, throughout pregnancy and to examine the differences between our two study groups. The predicted concentration of TGF-β in placental small EVs was significantly higher in the preeclampsia patients throughout pregnancy (P < 0.001) (Figure S6).

## Discussion

Our results showed that a pro-inflammatory state was present in the patients with preeclampsia, increasing the levels of pro-inflammatory cytokines and raising the pro- and anti-inflammatory cytokine ratios. The disruption of the balance between the pro- and anti-inflammatory cytokines in the peripheral blood was probably associated with the preeclampsia. These results supported by our findings that Th17 cytokines are dominant and contribute to maternal inflammation in preeclampsia. We established that the concentration of total and placental small EVs in the maternal circulation increased during pregnancy, in both normal and preeclamptic pregnancies. These results suggest that, in early pregnancy, presymptomatic women who are going to develop preeclampsia could be identified from the concentration of small EVs in their plasma.

In previous studies, little investigation of cytokines in EVs has been conducted. This study not only measured the level of cytokines in both plasma and placenta-derived small EVs, but also compared the level of cytokines between different trimesters. This study shows the potential role of small EVs in the pathophysiology of preeclampsia, providing biomarkers to predict the preeclampsia in early gestation. In our study, we used two markers to detect EVs for placental organ. The concentrations of total and placental small EVs in maternal circulation were quantified using CD63 and placental alkaline phosphatase (PLAP) enzyme-linked immunosorbent assay (ELISA) kits. In this study, we were to quantify plasma and then extract small EVs to detect cytokines, and there was no re-quantification of small EVs after extraction. Our total EVs included placenta small EVs, we detected EVs markers of placenta in total EVs. But we don't isolate specific placenta small EVs from total EVs in this study.

The pattern of cytokine release is not a fixed property of the system; it can be modulated, for example, upon maternal immune activation. However, due to restrictions in methodology or other unelucidated bioprocessing of the small EVs, there were inconsistent results for EV-encapsulated cytokines relative to the levels of plasma cytokines. Some EV-encapsulated pro-/anti-inflammatory cytokine ratios were reciprocal to the plasma ratios (Figure S7). These results may indicate the presence of another mechanism of small EVs in preeclampsia, involving a higher production of anti-inflammatory cytokines to suppress the immune response. Therefore, the dominance of pro-inflammatory cytokines is probably one of the most important factors affecting the pathogenesis of preeclampsia.

Dominance of pro-inflammatory cytokines appears to be harmful in pregnancy, and anti-inflammatory cytokines regulate and ameliorate the immune response 17, 18. The reports on which this statement is based measured the inflammatory biomarkers at only one time point during pregnancy, and obtained largely inconsistent findings in pregnant women with preeclampsia as a result of the variation in cytokine levels during gestation. We therefore measured the levels of cytokines in the plasma at several time points, to clarify the pattern throughout gestation. There is also an extensive and evolving literature on the equilibrium of Th17/Treg cytokines during pregnancy.

The Production of Tregs can induce by TGF-β, regulate the balance of Tregs/Th17, and is necessary to maintain the suppressive immune function of Tregs 19. TGF-β plays an important role in regulating the maternal-fetal interface to maintain immune homeostasis 20. On the other hand, TGF-β is upregulated and activated in fibrotic diseases and regulates fibroblast phenotype and function, inducing myofibroblast transdifferentiation 21. Another previous study indicated that TGF-β signaling has been implicated as a possible regulator of placental development and dysfunction 22. Furthermore, TGF-β is released in abundance by platelet activation that indicates possible mechanisms of platelet-trophoblast interactions and increased platelet activation on placenta development 23. Previous study was demonstrated that the mechanism underlying involved in an imbalance of Th17 and Treg in preeclampsia remained undefined, but there has been showed the significant relations between peripheral blood immune effectors and development of preeclampsia 24. Thus, the immunological assessments of peripheral circulation can be useful and less noninvasive in the prediction of preeclampsia and reduce the number of unsuccessful gestations 25.

Interestingly, impaired placental function in preeclamptic pregnancies results in placental apoptosis and necrosis, which further increases the release of small EVs 26, 27. Consistent with previous research, our study confirmed that the concentration of placenta-derived small EVs in the maternal circulation increased during gestation and was higher in preeclampsia patients than in healthy pregnant women 28. The bioactivity of placenta-derived small EVs may also exacerbate the pro-inflammatory state that is normally associated with pregnancy. With regard to cytokine segregation in small EVs and their biogenesis, small EVs seem to protect the encapsulated cytokines, as suggested by their stability relative to free-form cytokines in the peripheral circulation 11.

The effects of placenta-derived small EVs on both the fetus and the mother have yet to be clearly established. Elucidation of the small EVs pathways during normal and pathological pregnancies will allow the development of early screening tests for women at risk of pregnancy complications, monitoring of responses to interventions, and the development of novel EV-based therapies. There are certainly numerous new therapeutic possibilities involving small EVs, because their properties may be useful for the detection, amelioration, or curing of severe diseases. Furthermore, the bioactivity of placenta-derived small EVs may exacerbate the pro-inflammatory state that was normally associated with pregnancy 29. About cytokine segregation in small EVs and their biogenesis, small EVs seem to protect the encapsulated cytokines as shown by their stability, compared to free-form cytokines in the peripheral circulation 11. The release of cytokines either in a free-form or small EVs-associated form might reflect an adaptation to specific physiological needs, in particular these cytokines were needed to act near the secreting cell or at a distance 30. Thus, different biological systems differentially distributed the cytokines secreting within plasma and small EVs 11. The pattern of the cytokine released was not a fixed property of the system but rather can be modulated, for example upon maternal immune activation 31. However, due to the method restriction or other unelucidated mechanism of exosomal bioprocess, there were some inconsistent results in contrast to plasma cytokines. Some pro- / anti-inflammatory cytokine ratios were reciprocal to the results of those within plasma. These results would be indicated the other mechanism of small EVs in preeclampsia. There would be higher production of anti-inflammatory cytokines to suppress the immune response. Therefore, the dominance of pro-inflammatory cytokines was probably one of the important factors affecting preeclampsia pathogenesis. Nevertheless, this is one of the most robust studies conducted thus far to examine biomarkers of inflammation in pregnancy prospectively in relation to the development of preeclampsia.

## Conclusion

Our results demonstrate that changes in the levels of both plasma and EV-encapsulated cytokines in pregnant women with preeclampsia differed from those in healthy controls. The pro-inflammatory and anti-inflammatory cytokine profile shifted toward a pro-inflammatory state in the maternal peripheral circulation of the preeclampsia patients. In addition, both the plasma and EV-encapsulated cytokine ratios play an important role in the preeclampsia patients. The greatest differences in these biomarkers, even in the concentration of small EVs, between the two groups existed at the start of the second trimester. In addition, the abnormal concentration of total and placenta-derived small EVs may result in the secretion of numerous immunological effectors and be involved in a potential mechanism of immune regulation. Therefore, the plasma and EV-encapsulated cytokines, as well as their ratios, may be useful as non-invasive measurements for identifying women at high risk of preeclampsia during early gestation. However, a larger trial is required to further validate the utility of this approach for population screening. We look forward to constructing a novel prediction algorithm and improved guidelines for the identification of women at high risk of preeclampsia and the development of a personalized approach in the management of this condition.

## Supplementary Material

Supplementary figures and tables.

## Figures and Tables

**Table 1:** Comparison of the demographic characteristics of preeclampsia patients and healthy controls during the second trimester, and characteristics of their newborns

Characteristics	PE (N = 39)	HC (N = 127)	*P*
	Mean (SD)	Mean (SD)	
Age (years)	32 (5)	33 (4)	0.36
Height (cm)	161 (6)	160 (5)	0.76
Weight (kg)	71 (16)	61 (10)	< 0.01
BMI (kg/m^2^)	28 (7)	24 (4)	< 0.01
SBP (mmHg)	131 (17)	112 (13)	< 0.01
DBP (mmHg)	80 (14)	66 (8)	< 0.01
Gravidity	1.65 (0.82)	1.77 (0.93)	0.48
Parity	0.30 (0.52)	0.54 (0.68)	0.04
GA at delivery (weeks)	37 (3)	38 (1)	< 0.01
Birth weight (g)	2,771 (703)	3,060 (426)	0.01
Birth height (cm)	47 (3)	49 (2)	< 0.01
	**N (%)**	**N (%)**	
Alcohol consumption	7 (18)	28 (22)	0.66
Smoking	2 (5)	6 (5)	1.0
Primigravida	27 (69)	68 (54)	0.27
Previous preeclampsia	2 (5)	1 (1)	0.14
Thyroid dysfunction	1 (3)	4 (3)	1.0
Chronic hypertension	5 (13)	2 (2)	< 0.01
Fetal sex (male)	21 (54)	63 (51)	0.85
C-section	24 (62)	41 (33)	< 0.01
Preterm delivery	13 (33)	4 (3)	< 0.01

PE, preeclampsia patients; HC, healthy controls; BMI, body mass index; SBP, systolic blood pressure; DBP, diastolic blood pressure; GA, gestational age; C-section, Cesarean section; SD, standard deviation. *P*-values were estimated using Student's *t*-test or Fisher's exact test.

**Table 2:** Comparison of small EVs concentration and EV-encapsulated Th17/Treg cytokines in preeclampsia patients and healthy controls

	Small EVs (2^nd^ trimester)			Small EVs (3^rd^ trimester)		
	PE (N = 39)	HC (N = 127)		PE (N = 39)	HC (N = 127)	
Biomarkers	Median (IQR)	Median (IQR)	*P*	Median (IQR)	Median (IQR)	*P*
**EV markers**						
CD63	289.49 (130.92, 516.19)	178.39 (108.35, 219.35)	< 0.01	171.82 (119.36, 318.29)	146.40 (86.77, 214.27)	0.09
PLAP	234.32 (126.24, 425.05)	191.42 (139.77, 317.97)	0.42	202.82 (193.41, 264.87)	163.45 (60.88, 180.23)	< 0.01
**Th17**						
IL-17	4.60 (2.70, 10.58)	3.59 (1.74, 7.38)	0.51	3.01 (1.13, 6.05)	2.21 (1.42, 4.74)	0.56
IL-21	11.30 (7.21, 15.85)	3.83 (2.07, 8.59)	< 0.01	11.06 (7.91, 15.08)	7.55 (3.29, 11.80)	< 0.01
IL-22	12.50 (7.86, 17.65)	9.52 (5.16, 12.98)	< 0.01	11.29 (7.86, 15.62)	6.40 (2.69, 12.50)	< 0.01
**Treg**						
IL-10	2.57 (0.81, 3.59)	1.73 (0.92, 3.58)	0.41	1.31 (0.49, 4.43)	1.26 (0.53, 3.08)	0.59
TGF-β	13.56 (10.23, 20.17)	2.42 (0.59, 5.43)	< 0.01	7.27 (1.26, 13.15)	5.94 (2.46, 8.99)	0.40

EV, extracellular vesicles; PE, preeclampsia patients; HC, healthy controls; IL, interleukin; TGF, transforming growth factor; IQR, interquartile range; PLAP, placental alkaline phosphatase. *P*-values were estimated using the Wilcoxon rank-sum test.

**Table 3:** ROC curve analysis and support vector machine of EV biomarkers in preeclampsia patients and healthy controls during the second trimester

Biomarkers	ROC curve analysis			
	AUC (95% CI)	Accuracy (%)	Sensitivity (%)	Specificity (%)
**EV markers**				
CD63	0.77 (0.68, 0.86)	64	82	58
PLAP	0.71 (0.62, 0.80)	68	69	68
**Th17**				
IL-17	0.70 (0.61, 0.79)	73	64	75
IL-21	0.76 (0.67, 0.85)	70	72	70
IL-22	0.69 (0.60, 0.78)	64	72	62
**Treg**				
IL-10	0.64 (0.54, 0.73)	62	59	63
TGF-β	0.81 (0.72, 0.90)	73	74	73
**Multiple markers**				
CD63 + TGF-β	0.89 (0.84, 0.95)	81	90	78
PLAP + TGF-β	0.88 (0.81, 0.95)	81	87	80
IL-21 + TGF-β	0.87 (0.80, 0.94)	77	87	74
IL-17 + IL-21 + TGF-β	0.88 (0.81, 0.95)	79	87	76

ROC, receiver operating characteristic; EV, extracellular vesicles; AUC, area under the ROC curve; IL, interleukin; TGF, transforming growth factor; CI, confidence interval; PLAP, placental alkaline phosphatase.

## References

[1] Ananth CV, Keyes KM, Wapner RJ (2013). Pre-eclampsia rates in the United States, 1980-2010: age-period-cohort analysis. Bmj.

[2] Mol BW, Roberts CT, Thangaratinam S, Magee LA, De Groot CJ, Hofmeyr GJ (2016). Pre-eclampsia. Lancet.

[3] Qureshi ZP (2013). Moving beyond essential interventions for reduction of maternal mortality (the WHO Multicountry Survey on Maternal and Newborn Health): a cross-sectional study. Lancet.

[4] Many A, Schreiber L, Rosner S, Lessing JB, Eldor A, Kupferminc MJ (2001). Pathologic features of the placenta in women with severe pregnancy complications and thrombophilia. Obstet. Gynecol.

[5] Sebire N, Goldin R, Regan L (2005). Term preeclampsia is associated with minimal histopathological placental features regardless of clinical severity. J Obstet Gynaecol.

[6] Roberts JM, Bell MJ (2013). If we know so much about preeclampsia, why haven't we cured the disease?. J Reprod Immunol.

[7] Herrmann IK, Wood MJA, Fuhrmann G (2021). Extracellular vesicles as a next-generation drug delivery platform. Nat. Chem.

[8] Mincheva-Nilsson L, Baranov V (2014). Placenta-derived exosomes and syncytiotrophoblast microparticles and their role in human reproduction: immune modulation for pregnancy success. Am J Reprod Immunol.

[9] Guha D, Mukerji SS, Chettimada S, Misra V, Lorenz DR, Morgello S (2019). Cerebrospinal fluid extracellular vesicles and neurofilament light protein as biomarkers of central nervous system injury in HIV-infected patients on antiretroviral therapy. AIDS.

[10] Mihu D, Razvan C, Malutan A, Mihaela C (2015). Evaluation of maternal systemic inflammatory response in preeclampsia. Taiwan J Obstet Gynecol.

[11] Fitzgerald W, Freeman ML, Lederman MM, Vasilieva E, Romero R, Margolis L (2018). A system of cytokines encapsulated in extracellular vesicles. Sci Rep.

[12] Figueiredo AS, Schumacher A (2016). The T helper type 17/regulatory T cell paradigm in pregnancy. Immunology.

[13] Chang G-P, Yang X-L, Liu W, Lin S, Yang S-L, Zhao M-Y (2021). FABP4 facilitates inflammasome activation to induce the Treg/Th17 imbalance in preeclampsia via forming a positive feedback with IL-17A. Mol Ther Nucleic Acids.

[14] Darmochwal-Kolarz D, Kludka-Sternik M, Tabarkiewicz J, Kolarz B, Rolinski J, Leszczynska-Gorzelak B (2012). The predominance of Th17 lymphocytes and decreased number and function of Treg cells in preeclampsia. J Reprod Immunol.

[15] Lambrou A, Papadopoulos H, Nouretdinov I, Gammerman A (2012). Reliable probability estimates based on support vector machines for large multiclass datasets. IFIP International Conference on Artificial Intelligence Applications and Innovations: Springer.

[16] Furey TS, Cristianini N, Duffy N, Bednarski DW, Schummer M, Haussler D (2000). Support vector machine classification and validation of cancer tissue samples using microarray expression data. Bioinformatics.

[17] Saito S, Nakashima A, Shima T, Ito M (2010). Th1/Th2/Th17 and regulatory T-cell paradigm in pregnancy. Am J Reprod Immunol.

[18] Stefańska K, Zieliński M, Jankowiak M, Zamkowska D, Sakowska J, Adamski P (2021). Cytokine Imprint in Preeclampsia. FrontImmunol.

[19] Wang W-J, Hao C-F, Qu Q-L, Wang X, Qiu L-H, Lin Q-D (2010). The deregulation of regulatory T cells on interleukin-17-producing T helper cells in patients with unexplained early recurrent miscarriage. Hum. Reprod.

[20] Wang H, He M, Hou Y, Chen S, Zhang X, Zhang M (2016). Role of decidual CD14+ macrophages in the homeostasis of maternal-fetal interface and the differentiation capacity of the cells during pregnancy and parturition. Placenta.

[21] Pardali E, Sanchez-Duffhues G, Gomez-Puerto MC, Ten Dijke P (2017). TGF-β-induced endothelial-mesenchymal transition in fibrotic diseases. Int. J. Mol. Sci.

[22] Xu J, Sivasubramaniyam T, Yinon Y, Tagliaferro A, Ray J, Nevo O (2016). Aberrant TGFβ signaling contributes to altered trophoblast differentiation in preeclampsia. Endocrinol.

[23] Forstner D, Guettler J, Gauster M (2021). Changes in Maternal Platelet Physiology during Gestation and Their Interaction with Trophoblasts. Int. J. Mol. Sci.

[24] Lee JH, Ulrich B, Cho J, Park J, Kim CH (2011). Progesterone promotes differentiation of human cord blood fetal T cells into T regulatory cells but suppresses their differentiation into Th17 cells. J Microbiol Immunol Infect.

[25] Eghbal-Fard S, Yousefi M, Heydarlou H, Ahmadi M, Taghavi S, Movasaghpour A (2019). The imbalance of Th17/Treg axis involved in the pathogenesis of preeclampsia. J. Cell. Physiol.

[26] Redman C, Sargent I (2008). Circulating microparticles in normal pregnancy and pre-eclampsia. Placenta.

[27] Vargas A, Zhou S, Éthier-Chiasson M, Flipo D, Lafond J, Gilbert C (2014). Syncytin proteins incorporated in placenta exosomes are important for cell uptake and show variation in abundance in serum exosomes from patients with preeclampsia. FASEB.

[28] Salomon C, Guanzon D, Scholz-Romero K, Longo S, Correa P, Illanes SE (2017). Placental exosomes as early biomarker of preeclampsia: potential role of exosomal microRNAs across gestation. J Clin Endocrinol Metab.

[29] Jayabalan N, Nair S, Nuzhat Z, Rice GE, Zuñiga FA, Sobrevia L (2017). Cross talk between adipose tissue and placenta in obese and gestational diabetes mellitus pregnancies via exosomes. Front. Endocrinol.

[30] Desideri E, Ciccarone F, Ciriolo MR, Fratantonio D (2021). Extracellular vesicles in endothelial cells: from mediators of cell-to-cell communication to cargo delivery tools. Free Radic. Biol. Med.

[31] Mor G, Aldo P, Alvero AB (2017). The unique immunological and microbial aspects of pregnancy. Nat. Rev. Immunol.

