# Supplementary material for: Maternal Th17/Treg Cytokines and Small Extracellular Vesicles in Plasma as Potential Biomarkers for Preeclampsia
Source: Int J Med Sci. 2022 Sep 25;19(11):1672–9. doi: 10.7150/ijms.71047 (PMC9553855; doi:10.7150/ijms.71047)
Supplement: Supplementary file 1 — Supplementary figures and tables. [file ijmsv19p1672s1.pdf]

Table S1. Comparison of plasma Th17/Treg cytokines in preeclampsia patients and healthy controls

	Plasma (2nd trimester)			Plasma (3rd trimester)		
	PE (N = 39)	HC (N = 127)		PE (N = 39)	HC (N = 127)	
Cytokines	Median (IQR)	Median (IQR)	P	Median (IQR)	Median (IQR)	P
Th17						
IL-17	3.29 (1.44, 5.37)	2.01 (1.46, 3.22)	0.02	3.29 (1.92, 5.97)	2.52 (1.48, 3.54)	0.02
IL-21	17.25 (4.77, 39.84)	6.48 (2.82, 12.79)	< 0.01	26.94 (9.34, 63.89)	7.91 (2.96, 20.81)	< 0.01
IL-22	25.63 (13.15, 60.32)	12.14 (8.89, 20.88)	< 0.01	60.06 (22.46, 83.27)	11.63 (5.75, 20.88)	< 0.01
Treg						
IL-10	9.42 (4.42, 15.11)	6.49 (4.32, 9.87)	0.04	9.93 (6.93, 14.09)	4.36 (1.97, 8.49)	< 0.01
TGF-β	24.64 (10.92, 62.66)	18.09 (11.23, 23.85)	0.01	7.32 (2.37, 10.47)	7.16 (3.64, 12.01)	0.70

PE, preeclampsia patients; HC, healthy controls; IL, interleukin; TGF, transforming growth factor; IQR, interquartile range.

P-values were estimated using Wilcoxon rank-sum test.

Table S2. The pro-inflammatory and anti-inflammatory plasma cytokine ratios in preeclampsia patients versus healthy controls during the second trimester.

	Plasma (2nd trimester)		
	PE	HC	
Cytokine ratio	Median (IQR)	Median (IQR)	P-value
Th17/Treg			
IL-17/IL-10	0.40 (0.13, 0.82)	0.36 (0.18, 0.64)	0.75
IL-17/TGF-β	0.11 (0.04, 0.28)	0.13 (0.08, 0.25)	0.28
IL-21/IL-10	1.52 (0.74, 3.54)	1.04 (0.41, 2.28)	0.06
IL-21/TGF-β	0.58 (0.11, 1.62)	0.41 (0.15, 1.17)	0.55
IL-22/IL-10	3.99 (1.33, 7.43)	1.75 (1.03, 6.57)	0.08
IL-22/TGF-β	1.12 (0.46, 3.14)	0.82 (0.45, 1.87)	0.40

IL, interleukin; TGF, transforming growth factor; PE, preeclampsia; HC, healthy controls.

P values were estimated from Wilcoxon rank sum test.

Table S3. The pro-inflammatory and anti-inflammatory EV-encapsulated cytokine ratios in preeclampsia patients versus healthy controls during the second trimester.

	Small EVs (2nd trimester)		
	PE	HC	
Cytokine ratio	Median (IQR)	Median (IQR)	P-value
IL-17/IL-10	1.57 (0.60, 3.73)	2.12 (0.75, 4.04)	0.43
IL-17/TGF-β	0.36 (0.16, 0.48)	1.71 (0.52, 6.60)	< 0.01
IL-21/IL-10	4.16 (2.59, 9.03)	2.29 (1.45, 4.24)	< 0.01
IL-21/TGF-β	0.82 (0.27, 1.17)	1.64 (0.52, 5.59)	0.02
IL-22/IL-10	4.73 (2.89, 25.46)	5.23 (2.88, 9.34)	0.83
IL-22/TGF-β	1.01 (0.60, 1.43)	3.53 (1.29, 10.77)	< 0.01

IL, interleukin; TGF, transforming growth factor; PE, preeclampsia; HC, healthy controls.
P values were estimated from Wilcoxon rank sum test.

Table S4. ROC curve analysis and support vector machine of plasma cytokines in preeclampsia patients and healthy controls during the second trimester

		ROC curve analysis			Support vector machine			
Biomarkers	AUC (95% CI)	Accuracy (%)	Sensitivity (%)	Specificity (%)	Accuracy (%)	Sensitivity (%)	Specificity (%)	Cross-validation accuracy (%)
**Th17**								
IL-17	0.63 (0.52, 0.74)	58	67	55	81	79	81	74
IL-21	0.65 (0.56, 0.75)	57	64	55	76	56	82	72
IL-22	0.67 (0.57, 0.76)	61	72	58	83	79	84	75
**Treg**								
IL-10	0.58 (0.47, 0.68)	54	59	53	81	69	84	72
TGF-β	0.59 (0.48, 0.71)	55	62	54	84	82	85	77
**Multiple markers**								
IL-17 + IL-22	0.70 (0.60, 0.80)	60	64	59	80	77	81	66
IL-21 + IL-22	0.71 (0.62, 0.81)	66	72	65	83	69	87	73
IL-17 + IL-21 + IL-22	0.72 (0.62, 0.82)	60	72	56	91	87	92	70

ROC, receiver operating characteristic; AUC, area under the ROC curve; IL, interleukin; TGF, transforming growth factor; CI, confidence interval.

Table S5. Support vector machine of plasma cytokine ratios in preeclampsia patients and healthy controls during the second trimester.

Cytokine ratio	Support vector machine			
	Accuracy (%)	Sensitivity (%)	Specificity (%)	Cross-validation accuracy (%)
IL-17/IL-10	77	62	81	65
IL-17/TGF-β	72	58	76	64
IL-21/IL-10	72	38	82	66
IL-21/TGF-β	85	63	91	73
IL-22/IL-10	73	49	81	69
IL-22/TGF-β	78	58	84	65
**Multiple markers**				
IL-21/IL-10 + IL-22/IL-10	79	64	83	65
IL-21/IL-10 + IL-22/IL-10 + IL-22/TGF-β	86	100	82	62

ROC, receiver operating characteristic; IL, interleukin; TGF, transforming growth factor; AUC, area under the ROC curve; CI, confidence interval.

Table S6. Support vector machine of EV-encapsulated cytokine ratios in preeclampsia patients and healthy controls during the second trimester.

Cytokine ratio	Support vector machine			
	Accuracy (%)	Sensitivity (%)	Specificity (%)	Cross-validation accuracy (%)
IL-17/IL-10	83	81	84	61
IL-17/TGF-β	94	96	92	82
IL-21/IL-10	69	75	66	62
IL-21/TGF-β	77	73	81	66
IL-22/IL-10	73	61	79	55
IL-22/TGF-β	71	81	64	64
**Multiple markers**				
IL-17/TGF-β + IL-22/TGF-β	85	82	88	70
IL-17/TGF-β + IL-21/TGF-β + IL-22/TGF-β	86	75	96	80

ROC, receiver operating characteristic; IL, interleukin; TGF, transforming growth factor; AUC, area under the ROC curve; CI, confidence interval.

The best cut-off point of IL-17/TGF-β is < 0.63.

The best cut-off point of IL-22/TGF-β is < 0.52.

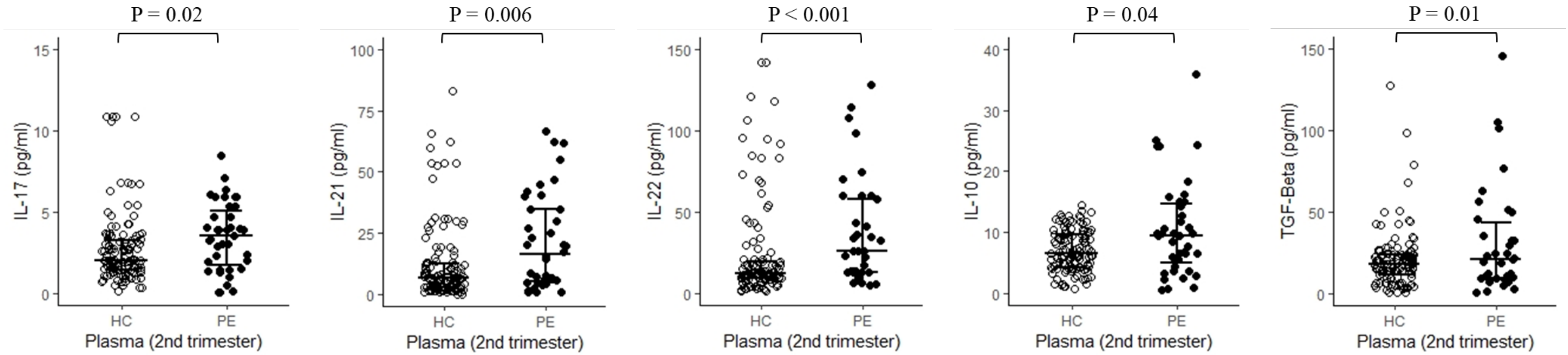

Figure S1. Plasma levels of Th17 and Treg cytokines between preeclampsia (PE) patients and healthy controls (HC) in the second trimester. Data are presented as an aligned dot plot, and values are median and quartiles. The p values were calculated by Wilcoxon rank sum test.

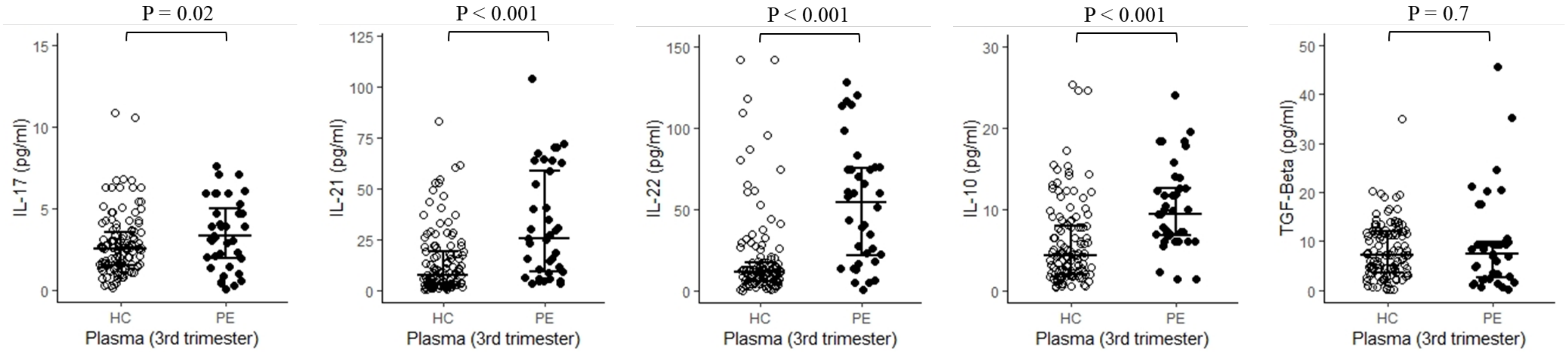

Figure S2. Plasma levels of Th17 and Treg cytokines between preeclampsia (PE) patients and healthy controls (HC) in the third trimester.

Data are presented as an aligned dot plot, and values are median and quartiles. The p values were calculated by Wilcoxon rank sum test.

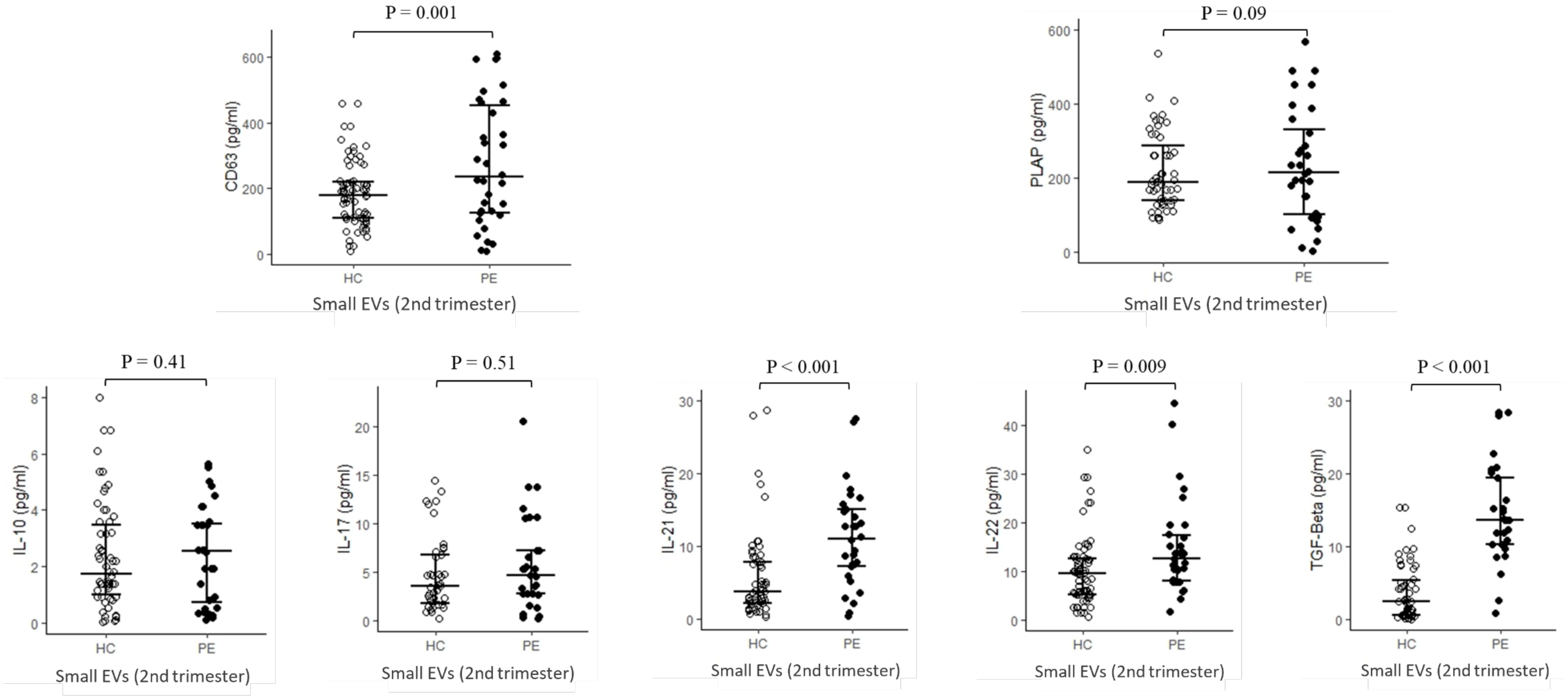

Figure S3. Total concentration of Th17 and Treg cytokines and placental-derived small EVs concentration of Th17 and Treg cytokines between preeclampsia (PE) patients and healthy controls (HC) in the second trimester.

Data are presented as an aligned dot plot, and values are median and quartiles. The p values were calculated by Wilcoxon rank sum test.

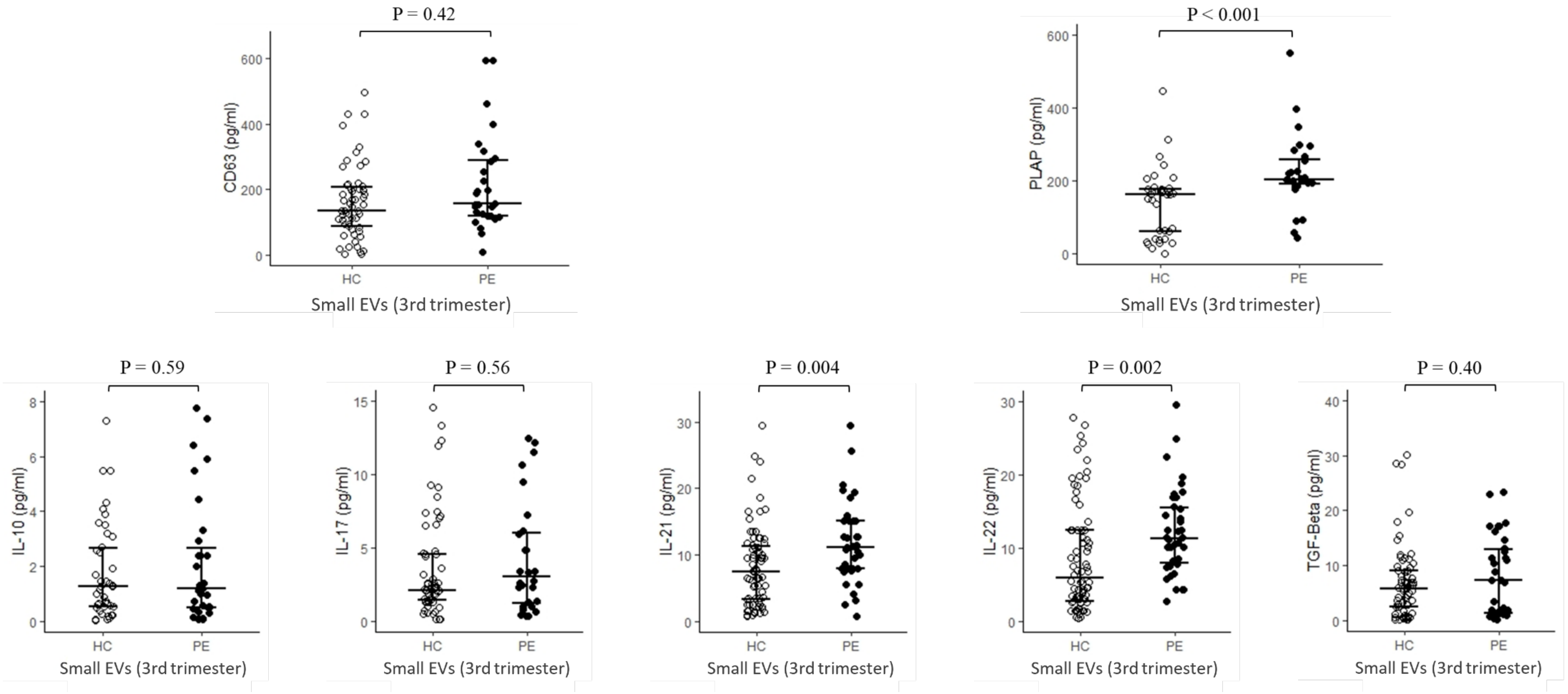

Figure S4. Total concentration of Th17 and Treg cytokines and placental-derived small EVs concentration of Th17 and Treg cytokines between preeclampsia (PE) patients and healthy controls (HC) in the third trimester.

Data are presented as an aligned dot plot, and values are median and quartiles. The p values were calculated by Wilcoxon rank sum test.

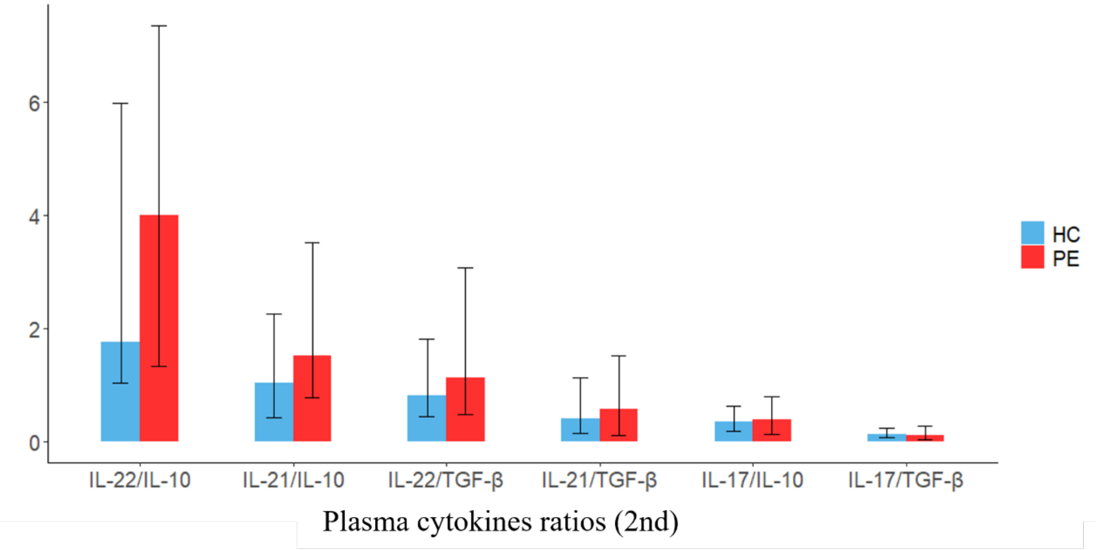

Figure S5. Plasma cytokine ratios between preeclampsia patients and healthy controls during the second trimester. Data are presented with medians and quartiles of each cytokine in bar charts.

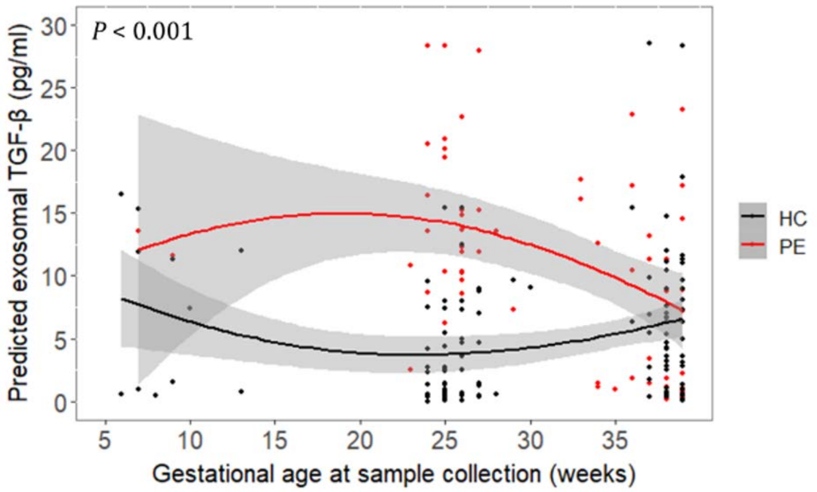

Figure S6. Predicted values of EV-encapsulated TGF-β from the generalized additive mixed models, in relation to Loess-smoothed gestational age at sample collection, in preeclampsia patients (red points and lines) and healthy controls (black points and lines). Solid lines represent predicted values; grey areas represent the 95% confidence interval for each group. Points represent the actual biomarker concentrations.

TGF-β, transforming growth factor beta

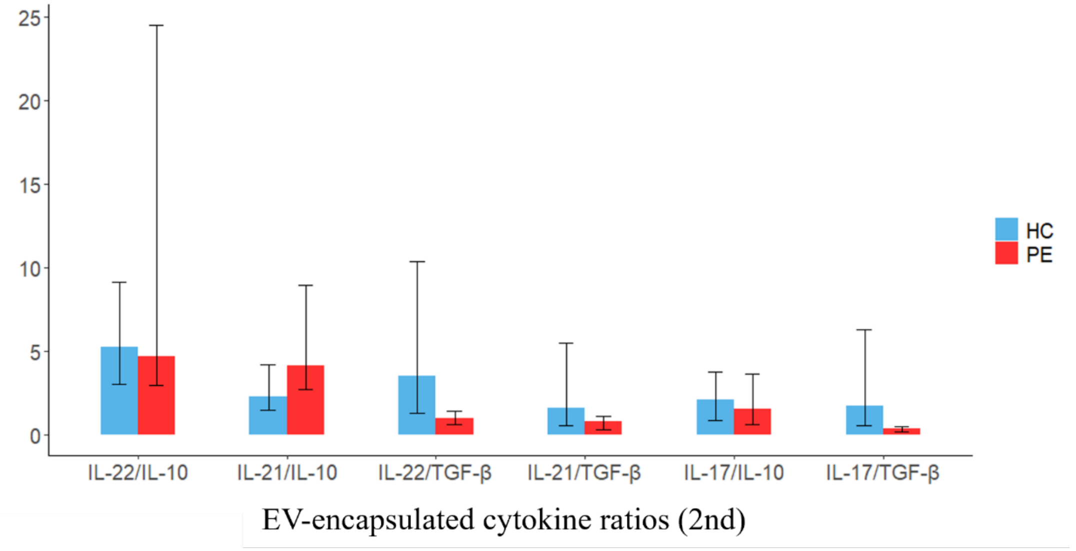

Figure S7. EV-encapsulated cytokine ratios between preeclampsia patients and healthy controls during the second trimester. Data are presented with medians and quartiles of each cytokine in bar charts.
